# Light interacts with mechanical stress to regulate the seed-to-seedling transition

**DOI:** 10.1007/s44154-025-00269-y

**Published:** 2025-12-30

**Authors:** Yun Meng, Jiashuai Wu, Javed Iqbal, Shameen Sajid, Qingqing Wu

**Affiliations:** National Engineering Laboratory of Crop Stress Resistance Breeding, School of Life Sciences, Anhui Agricultural University, Hefei, 230036 China

**Keywords:** Light, Mechanical stress, Seed-to-seedling, Deep sowing

## Abstract

The transition from seed to seedling represents a critical developmental phase that determines seedling survival, crop establishment, and yield potential. This intricate developmental process encompasses multiple stages: seed germination beneath the soil surface, the upward growth of etiolated seedlings through the soil environment to reach the soil surface, and subsequent greening to support photoautotrophic growth. The key environmental factors influencing the transition of buried seed to seedling establishment are light, mechanical resistance imposed by soil cover, and the intricate interplay between these factors. Recent studies have significantly enhanced our comprehension of the dynamic and complex nature of this transition: as a seedling pushes upward through the soil, light exposure steadily increases while mechanical resistance gradually decreases. In response, seedlings must orchestrate the initiation of light-regulated developmental processes with adjustments to mechanical stress. This review summarizes the molecular mechanism through which light and mechanical stress interact to facilitate and optimize the transition from seed to seedling in *Arabidopsis*, with a particular emphasis on deep sowing conditions in rice and maize. Insights into these molecular mechanisms can advance our understanding of the seed-to-seedling biology and contribute to the genetic improvement of crops.

## Introduction

Crop seeds are typically sown into the soil, where they initiate their life cycles under favorable conditions, enabling optimal adaptation to the environment and achieving high fitness (Shu et al. 2016; Nonogaki 2019). Beneath the soil, healthy dry seeds imbibe water, and break through the constraints of the seed coat to achieve germination (Carrera-Castano et al. 2020). Subsequently, under the influence of the soil cover, post-germinative seedlings undergo an adaptive etiolated growth phase in darkness, termed skotomorphogenesis (Von Arnim and Deng 1996; de Wit et al. 2016). This particular phase is distinguished by rapid hypocotyl elongation and apical hook bending, which facilitate the seedlings’ emergence from the soil surface while protecting the cotyledons from mechanical damage (An et al. 2012). As etiolated seedlings grow upward into the light, they undergo a dramatic photomorphogenic developmental transition, marked by the inhibition of hypocotyl elongation and the unfolding of closed cotyledons. Successfully turn greening or de-etiolation, chlorophyll biosynthesis and chloroplast development, which allow seedlings to swiftly commence photoautotrophic growth while avoiding photooxidation, must be promptly and strictly controlled (Briggs 2016; Von Arnim and Deng 1996; Chen et al. 2004; Huq et al. 2024; Zhong et al. 2014; Wang and Guo 2019). All of these transitions mark the successful seedling establishment (Venkateswara et al. 2021).

The crucial light signals that initiate this vital seedling establishment are primarily detected by different type of photoreceptors that are capable of monitoring the entire light spectrum spanning from ultraviolet-B wavelengths to far-red and near-infrared wavelengths (Jiao et al. 2007). These photoreceptors facilitate the transduction of light signals by engaging in protein–protein interactions with key transcription factors or E3 ligases, thereby governing the stability of crucial regulatory proteins. The basic helix-loop-helix (bHLH) family transcription factors, known as PHYTOCHROME-INTERACTING FACTORS (PIFs), along with the plant-specific transcription factors ETHYLENE-INSENSITIVE 3 (EIN3) and EIN3-LIKE 1 (EIL1), play a negative regulatory role in the light signaling pathway (Ni et al. 1998; Leivar and Monte 2014; Pham et al. 2018; Cai et al. 2025). These transcription regulators orchestrate the overall transcriptional program to facilitate the execution of light signaling cascades (Jing and Lin 2020). CONSTITUTIVE PHOTOMORPHOGENIC1 (COP1) serves as a pivotal negative regulator of the plant light signaling pathway. It functions downstream of diverse photoreceptors and facilitates the degradation of positive regulators in light signal pathway via the 26S-proteasome pathways (Lau and Deng 2010; Hardtke et al. 2000). ELONGATED HYPOCOTYL5 (HY5) and its close homolog HY5 HOMOLOG (HYH) are a pair of basic leucine zipper transcription factors (bZIPs) that are crucial in suppressing hypocotyl elongation and serve as key positive regulators within light signaling pathway (Oyama et al. 1997; Holm et al. 2002; Chen et al. 2013).

Seed-to-seedling transition not only undergoes intricate light-mediated developmental procedures but also confronts the mechanical stress imposed by complex soil conditions (Gupta and Nath 2020). Over the past few years, the significant role of light in regulating seed germination, as well as etiolated and de-etiolated growth has been widely revealed. However, the precise mechanism through which seedlings perceive soil depth and subsequently adjust their growth and development remains largely elusive. In recent years, by utilizing real-soil assays, we have begun to make substantial progress in unraveling how mechanical stress signaling pathways modulate seed-to-seedling transition and how they interact with light signals (Zhong et al. 2014). Here, we present a comprehensive overview of current understanding concerning the intricate interplay between light and mechanical stress, which orchestrates the transition from seed germination to the thriving-establishment of seedlings, especially in crops subjected to deep sowing conditions. Finally, we outline the research prospects in this field, with a focus on mechanistic investigations and the potential application of these findings in breeding varieties suitable for deep-sowing.

## Seed germination

**Fig. 1:**
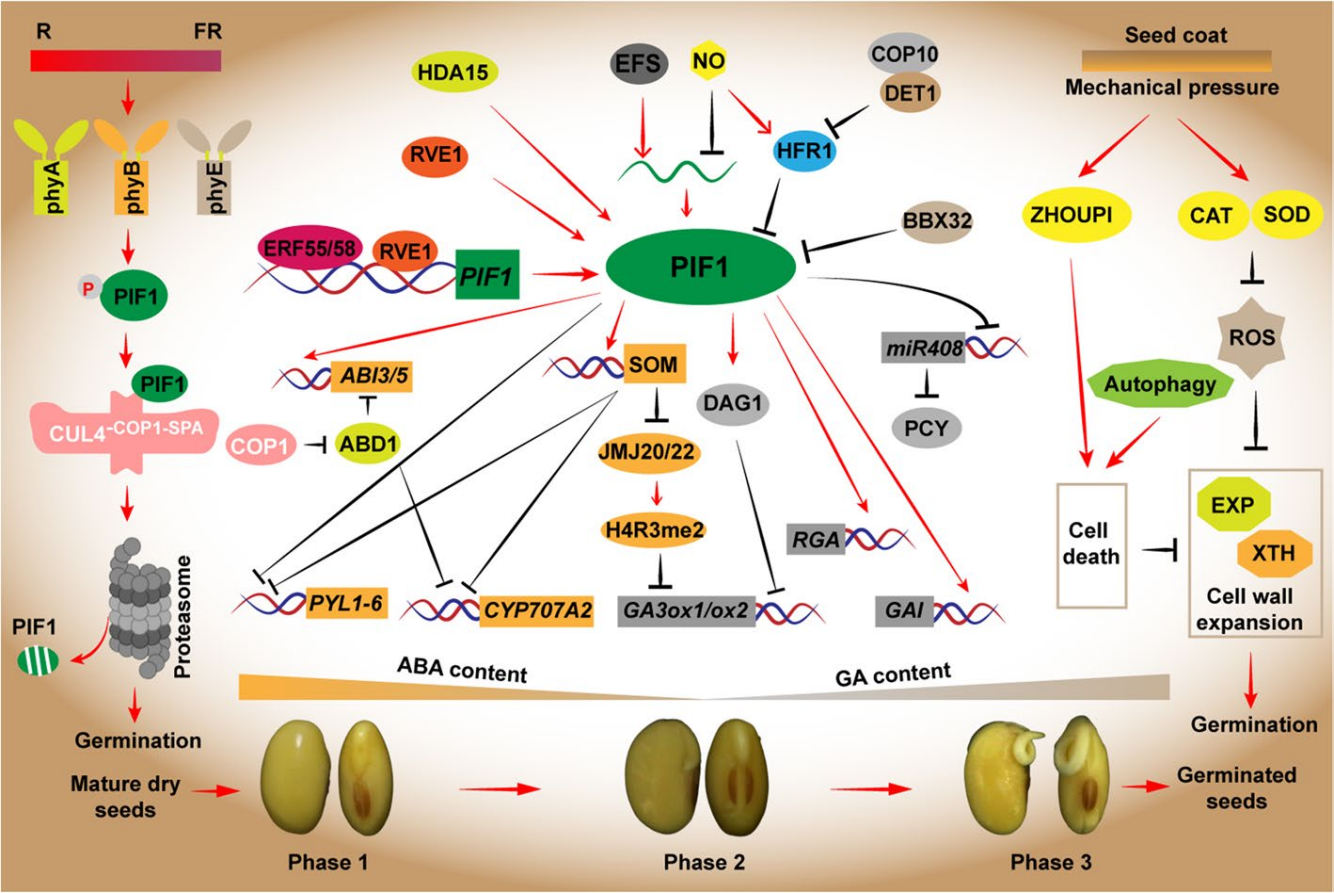
Light and mechanical interplay regulate seed germination. ABA, Abscisic acid; GAs, Gibberellins. Arrows and lines with slanted dashes indicate positive and negative effects, respectively

Nondormant seeds monitor their surrounding environment to precisely orchestrate the germination process, as germination determines the crucial step toward seed-to-seedling establishment and serves as the basis for crop yield (de Wit et al. 2016; Zhao et al. 2024a). The germination of a seed is marked by three distinct water uptake phases: Phase 1, initial imbibition; Phase 2, a subsequent lag period; and Phase 3, a final phase of active water absorption driven by growth (Finch-Savage and Leubner-Metzger 2006). The germination procedure is governed by multiple environmental factors, including temperature (Kendall et al. 2011; He et al. 2014), oxygen availability (Abbas et al. 2015), and nutrient supply (Footitt et al. 2013). Light serves as another crucial environmental factor that initiates the germination process (de Wit et al. 2016; Yang et al. 2020b). This review centers the effects of light-mediated seed germination (Fig. 1).

### Roles of photoreceptors-phytochromes in seed germination

The roles of phytochromes in regulating seed germination were first identified in lettuce. By quantifying the spectrum, Borthwick et al. discovered that red light promotes germination, while far-red light inhibits it (Borthwick et al. 1952). A parallel response has been revealed observed in *Arabidopsis* (Shinomura 1997; Hennig et al. 2002). Hence, for light-requiring seeds, the red to far-red (R/FR) ratio is utilized to determine the suitability of the light environment and to determine the optimal timing for germination. The germination of *Arabidopsis* seeds is regulated by a group of phytochromes, including phyA, phyB, and phyE, within hours of imbibition. PhyA, which is sensitive to both very low and high fluence responses to FR light, to promote seed germination. In contrast, phyB plays a dominant role in the photo-reversible reaction triggered by R/FR light, to regulate germination (Merai et al. 2023; Dechaine et al. 2009). On the other hand, phyE stimulates germination under continuous FR light (Hennig et al. 2002) and at very low R/FR ratio (Arana et al. 2014). Thus, phytochromes exhibit redundant effects on germination process, with phyB assuming a predominant role throughout the germination phase.

### Light-mediated degradation of PIF1 promotes light-induced seed germination

Under dark or FR light conditions, PIF1 accumulates abundantly in seeds, where it suppresses the expression of germination-related genes. Upon light exposure, phytochromes interact with PIF1, triggering its phosphorylation, which subsequently results in its degradation through the CUL4^−COP1−SPA^ E3 ligase complex (Xu et al. 2014), thereby alleviating PIF1’s inhibitory effects and initiating light-induced germination process (Shen et al. 2005). In darkness, COP1 promotes the post-translational stability of ABI5 to regulate germination, which is achieved by destabilizing ABA-HYPERSENSITIVE DDB1-CUL4-ASSOCIATED FACTOR 1 (ABD1), a substrate receptor that normally targets ABI5 for degradation (Peng et al. 2022).

The transcriptional activity and protein stability of PIF1 are jointly controlled by multiple pathways. Numerous members of the ETHYLENE RESPONSE FACTOR (ERF) transcription factor family in plants, such as ERF55 and ERF58, interact with phytochromes and directly bind to the promoter region of *PIF1* and activate its transcription (Li et al. 2022). REVEILLE1 (RVE1) and PIF1 not only interact but also directly bind to the promoters of each other, forming a transcriptional feedback loop that reciprocally activate their expression (Yang et al. 2020a). The red light-induced transcription factor LONG HYPOCOTYL IN FAR-RED1 (HFR1) interacts with PIF1 and assists in regulating the transcription of downstream genes to promote germination (Shi et al. 2013). As an upstream interacting protein of both HFR1 and PIF1, DE-ETIOLATED1 (DET1) promotes HFR1 degradation while enhancing PIF1 stability through two independent signaling pathways: the phyB-DET1-HFR1-PIF1 pathway and the phyB-DET1-protease-PIF1 pathway. Concurrently, COP10 promotes HFR1 degradation by forming the COP10-DET1-DDB1-CUL4 E3 ubiquitin ligase complex, thereby releasing PIF1’s transcriptional activity (Shi et al. 2013).

The nitric oxide (NO), as signaling molecule inhibits PIF1 transcription while stabilizing HFR1, promoting the formation of the HFR1-PIF1 heterodimer (Li et al. 2018). The histone deacetylase HDA15 interacts with PIF1 in the dark, reducing histone H3 acetylation levels and inhibiting germination (Gu et al. 2017). The histone H3K4/H3K36 methyltransferase EARLY FLOWERING IN SHORT DAYS (EFS) directly targets *PIF1* locus, enhancing *PIF1* expression and inhibiting germination by increasing H3K36me2 and H3K36me3 modifications (Lee et al. 2014). Current studies demonstrates that water-imbibition-activated B-BOX DOMAIN PROTEIN 32 (BBX32) directly associates with PIF1, disrupting its self-interaction and DNA-binding ability, while acting in parallel with HFR1 to alleviate the supressive role of PIF1 on germination (Gao et al. 2024).

PIF1 exerts a potent inhibitory influence on seed germination in the dark via orchestrating the transcription of genes involved in gibberellin (GA) and abscisic acid (ABA) signaling. PIF1 negatively regulates GA signaling by directly inducing the expression of two DELLA genes, *GIBBERELLIC ACIDINSENSITIVE* (*GAI*) and *REPRESSOR OF GA1-3* (*RGA*) (Li et al. 2016). At low R/FR ratios, PIF1 activates *DOF AFFECTING GERMINATION1* (*DAG1*), which indirectly inhibits GA biosynthesis by blocking *GA3ox1* expression, thereby suppressing seed germination (Boccaccini et al. 2014). Additionally, PIF1 directly binds to the promoters of the key ABA signaling transcription factors *ABSCISIC ACID INSENSITIVE 3* and *5* (*ABI3/5*) (Liew et al. 2024; Qi et al. 2020), along with the negative regulator *SOMNUS* (*SOM*), and activates their transcription (Kim et al. 2021). *SOM*, in a regulatory feedback loop, suppresses the activity of *JUMONJI20* (*JMJ20*) and *JMJ22*, leading to elevated levels of H4R3me2 at the chromatin of *GA3ox1* and *GA3ox2*, ultimately repressing their expression (Cho et al. 2012; de Wit et al. 2016). Meanwhile, PIF1 and SOM1 suppress GA biosynthesis by inhibiting the transcription of GA synthesis genes *GA3ox1* and *GA3ox2* and promoting the transcription of the GA catabolic gene *GA2ox2*, which is involved in GA inactivation (Kim et al. 2021; Li et al. 2022; Oh et al. 2006). Simultaneously, accumulation of PIF1 and SOM inhibit the expression of ABA receptors *PYL1-6* and the ABA catabolic gene *CYP707A2*, resulting in increased ABA content and inhibition of germination (Li et al. 2022). Moreover, PIF1 interacts with the promoter of the small RNA molecule *miR408*, suppressing its accumulation, which in turn regulates the abundance of *PLANTACYANIN* (*PCY*). This ultimately reduces the GA content in the embryo and inhibits germination (Jiang et al. 2021).

### Mechanical interplay in seed germination

The mechanical constraints imposed by seed/fruit coats and endosperm are critical factors that influence the initiation of germination. The decrease in cell wall mechanical strength in these “coats” facilitates embryo growth and radicle protrusion. For example, the transcription factor ZHOUPI triggers cell death and modifies the mechanical properties of the endosperm cell wall, enabling the embryo to exert pressure on the surrounding endosperm (Lee et al. 2014; Yang et al. 2008). At the germination process, cell differentiation and expansion are accompanied by key regulators, such as XYLOGLUCAN ENDOTRANSGLUCOSYLASEs/HYDROLASES (XTHs), α-EXPANSINS (EXPs), pectin-modifying enzymes, and intracellular reactive oxygen species (ROS). These factors collectively mediate cell wall loosening and expansion (Bashline et al. 2014; Karkonen and Kuchitsu 2015). Robust respiratory activity and energy metabolism lead to a gradual increase in intracellular ROS levels, triggering an antioxidant response mediated by antioxidant enzymes including catalase (CAT) and superoxide dismutase (SOD), thereby maintaining intracellular ROS homeostasis and ensuring the successful germination (Xi et al. 2010). Furthermore, autophagy plays a key role in preserving endosperm viability throughout seed storage by preventing senescence-induced oxidative destruction and cell death, ensuring that the endosperm functions optimally for subsequent germination (Shinozaki et al. 2024).

## Seedling establishment

**Fig. 2:**
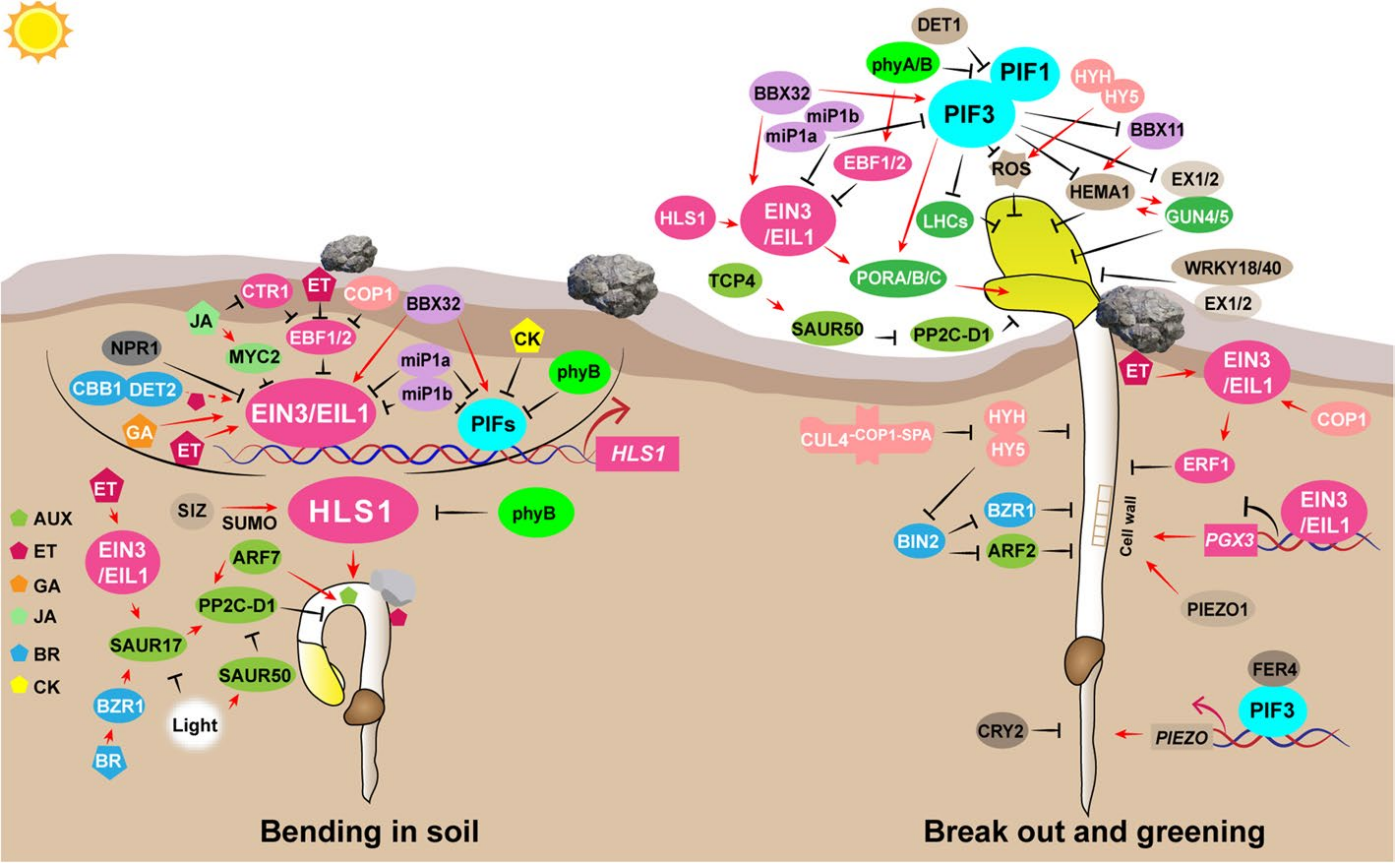
Light interacts with mechanical stress to regulate the seed to seedling establishment. Aux, Auxin; ET, Ethylene; GAs, gibberellins; JA, Jasmonic acid; BRs, Brassinosteroids; CK, Cytokinin. Arrows and lines with slanted dashes indicate positive and negative effects, respectively

In the preceding section, we discussed how seeds germinate under light conditions. However, many seeds are buried in soil, and these seedlings must dynamically modulate their growth patterns in response to the encroaching darkness and the mechanical constraints imposed by the soil barrier. In this section, we concentrate on the molecular mechanisms governing the regulation of seedling establishment, including apical hook development, hypocotyl growth, and the greening process, which involve the intricate interactions between light signals and mechanical stress caused by the soil overlay (Fig. 2).

### Bending in the soil: apical hook development

For most of dicotyledonous seedlings, the formation and termination of the apical hook are critical for progressive and timely seedling emergence (Shen et al. 2016; Bai 2021; Wang et al. 2023b). Current research have revealed that the formation and termination of the apical hook are orchestrated by an intricate signaling network integrating multiple plant hormones and light signals, converging on the key controller of apical hook development: HOOKLESS1 (HLS1) (Fig. 2).

Phytohormones and their interactions shows a crucial role in menapulating the development of the apical hook (Wang And Guo 2019; Wang et al. 2023b). The development progression of the apical hook has been extensively defined as an anisotropic growth process: the lateral, asymmetric distribution of auxin in the apical part of the hypocotyl drives hook development (Wang And Guo 2019; Abbas et al. 2013; Jonsson et al. 2021). Latest findings have demonstrated that an increase in auxin concentration in the interior side of the apical hook activates *AUXIN RESPONSE FACTOR 7* (*ARF7*), thereby triggering the expression of *PHOSPHATASE 2 C D-CLADE 1* (*PP2C-D1*). PP2C-D1 subsequently dephosphorylates and inhibits plasma membrane H^+^-ATPases, thereby blocking H^+^ efflux, causing apoplastic alkalinization, and inhibiting cell expansion along the inner hook (Du et al. 2022; Tang et al. 2025; Smetana et al. 2019). The ethylene signaling cascade components EIN3/EIL1 modulate the transcription of *HLS1* by directly binding to its promoter (An et al. 2012). GA induces *HLS1* expression through an EIN3/EIL1-dependent mechanism (An et al. 2012). Brassinosteroids (BRs) are essential for etiolation, as mutants with impaired BR synthesis or signaling fail to develop an apical hook and exhibit open cotyledons (Li et al. 1996). Furthermore, in BR biosynthesis-deficient mutants, *de-etiolated2* (*det2*) and *cabbage1* (*cbb1*), apical hook formation is impaired, and exogenous ethylene cannot restore the apical hook phenotype in these mutants (Wei et al. 1994; Jiroutova et al. 2018). Ethylene (ET) and BRs can stimulate apical hook formation by activating the *SMALL AUXIN UP RNA17* (*SAUR17)* gene expression (Wang et al. 2023a). In addition, cytokinins (CKs) positively regulate the development of apical hooks by increasing the protein levels of light-sensitive transcription factors PIFs (Aizezi et al. 2022). In contrast, JA inhibits apical hook formation, and when both JA and ET are applied together, JA attenuates the promoting effect of ethylene on hook development (Zhang et al. 2018). In the *constitutive triple response1* (*ctr1*) mutant, the application of JA effectively suppresses the exaggerated hook phenotype. Recent studies have revealed that JA activates the MYB family transcription factor MYC2, which not only negatively regulates EIN3 but also directly interacts with it, thereby exerting both direct and indirect inhibitory effects on the EIN3-mediated transcriptional activation of *HLS1* (Aizezi et al. 2022). The NPR1 protein, a crucial component in the SA signaling pathway, can directly interact with the EIN3 protein and inhibit the binding of EIN3 to the promoters of its downstream target gene *HLS1*, consequently inhibiting the formation of apical hooks (Huang et al. 2020).

Apart from plant hormones, light also serves as a pivotal regulator in the development of the apical hook. Under light conditions, phyB interacts directly with HLS1, disrupting its self-oligomerization and changing it into a monomeric state. This light-induced transition attenuates HLS1 activity, promotes apical hook opening, and facilitates successful seedling emergence (Lyu et al. 2019). PIFs collaborate with EIN3/EIL1 to enhance hook formation by stimulating the transcription of *HLS1*. They regulate the development of the apical hook via both independently and cooperatively by modulating a combination of shared and distinct target genes (Zhang et al. 2018). SUMOylation is essential for HLS1 function, as it promotes the assembly of HLS1 into its active oligomeric form. During de-etiolation, light triggers apical hook opening, accompanied by reduced transcription of small ubiquitin-like modifier (SUMO) E3 ligase SAP AND MIZ1 DOMAIN-CONTAINING LIGASE1 (SIZ1) and lower HLS1 SUMOylation (Xiong et al. 2023). Moreover, EIN3-BINDING F-BOX PROTEIN 1/2 (EBF1/2) are directly targeted for ubiquitination and subsequent degradation by COP1, thereby stabilizing EIN3 and contributing to enhance apical hook formation (Osterlund et al. 1999; Shi et al. 2016a). Intriguingly, light signals downregulate *SAUR17* while upregulating *SAUR50* within the inner cells of the apical hook and cotyledon, with antagonistic regulation of PP2C-D1, which promotes cell expansion, resulting in the unfolding of the apical hook and cotyledons (Wang et al. 2020). MicroProteins (miPs), as newly identified translation-regulating factors, also play roles in light-mediated hook opening. Plants overexpressing miP1a or miP1b are able to overcome the delayed hook opening observed in PIF3ox or EIN3ox plants. Consequently, miP1a and miP1b promote the transition to photomorphogenesis by accelerating cotyledon unfolding (Wu et al. 2020a). Recently, ET and light were shown to cooperatively act on BBX32 to positively regulate *HLS1* expression by enhancing PIF3 binding to the *HLS1* promoter, thereby inhibiting apical hook opening and promoting soil emergence (Ravindran et al. 2025).

### Break out: hypocotyl elongation regulation

In dicotyledonous seedlings, the hypocotyl is one of the core organs during the seedling establishment phase. Its elongation, growth orientation, and response to soil pressure directly determine whether seedlings can successfully survive in the soil (Fig. 2).

Soil overlay significantly elevates the abundance of EIN3/EIL1 protein, which plays a vital role in stimulating hypocotyl elongation as seedling emerge from the soil (Zhong et al. 2014). During soil emergence, accumulated ethylene in seedlings couples two pathways for successful soil emergence, activating ERF1 cascade to inhibit cell growth and activating PIF3 pathway to promote greening (Zhong et al. 2014; Shen et al. 2016; Solano and Ecker 1998). Mechanical stimuli can regulate the molecular weight of pectin polymers and polygalacturonase (PG) activity in etiolated seedlings, which is linked to EIN3 directly binding to the *POLYGALACTURONASE INVOLVED IN EXPANSION* (*PGX3*) promoter in a dose-dependent manner, rapidly suppressing *PGX3* transcription and PG activity and facilitating seedling soil emergence (Wu et al. 2020b).

In addition, FERONIA (FER), a receptor-like kinase can sense changes in cell wall status, such as lignin reduction or pectin expansion, facilitating the degradation of components like pectin and the release of oligosaccharides, and plays a key role in hypocotyl elongation and environmental adaptation (Li et al. 2023). *AtPIEZO1*, the homolog of moss *PIEZO*, is also localized to the vacuolar membrane of hypocotyl and petiole cells, and it affects hypocotyl growth and development by regulating vacuole morphology and calcium ion oscillations (Radin et al. 2021). FER4 interacts with the root-localized mechanosensitive ion channel PIEZO via PIF3 to sense soil confinement and regulate the ability to penetrate (Xu et al. 2024).

In darkness or when buried in soil, COP1 promotes skotomorphogenic growth characteristics by associating with SPA proteins that facilitate the ubiquitination and degradation of transcription factor like HY5 (Han et al. 2020; Wang et al. 2019). This COP1-HY5 module maintains skotomorphogenic traits, such as etiolated and elongated hypocotyls (Jing and Lin 2020; Mankotia et al. 2024). However, HY5 trigger transcription expression by directly interacting to cis-acting elements in target gene promoters, such as the G-box, Z-box, and others, which play pivotal roles in repressing seedling hypocotyl elongation (Peng et al. 2022; Oyama et al. 1997; Holm et al. 2002; Lee et al. 2007; Zhang et al. 2011; Li et al. 2020; Ang et al. 1998). The physical interaction between HY5 and other transcription factors directly regulates its transcriptional output, that can form heterodimers with HY5 (Singh et al. 2012; Holm et al. 2002). In addition, HY5 can enhance BRASSINOSTEROID-INSENSITIVE (BIN2) kinase activity through physical interaction, thereby promoting BIN2-mediated phosphorylation and degradation of BRASSINAZOLE-RESISTANT1 (BZR1), which represses BR-mediated hypocotyl elongation in the light (Li et al. 2020). BR-regulated BIN2 kinase directly phosphorylates ARF2, thereby inactivating the repressor ARF2 and increasing the expression of auxin-induced genes to enhance hypocotyl elongation (Vert et al. 2008). In addition, the non-photoconverted form of CRYPTOCHROME 2 (CRY2) inhibits root elongation and directs the limited energy of the seed toward hypocotyl elongation, aiding seedling soil emergence and the initiation of photosynthetic autotrophy (Zeng et al. 2025).

### Successful greening

Once seedlings emerge from the soil and are exposed to light, they initiate the process of greening, during which they must finely tune their growth patterns and orchestrate the development of photosynthetic organs accordingly (de Wit et al. 2016). Protochlorophyllide (Pchlide), a predecessor of chlorophyll that accumulates in subterranean seedlings (Hiratsuka And Chua 1997; Liu et al. 2017), can cause photooxidative damage or even death of seedlings, when excessive accumulation occurs and cannot be fully converted into functional chlorophyll (Reinbothe et al. 2010; op den Camp et al. 2003; Li et al. 2023; Wang et al. 2025). Therefore, the greening process is a crucial stage in the entire lifecycle of terrestrial plants, determining whether plants survive successfully (Fig. 2).

Plants have evolved complex and sophisticated molecular mechanisms that regulate Pchlide biosynthesis. EIN3, which functioning downstream of COP1, plays a vital role in promoting seedling greening by directly binding to the promoter regions of the *PROTOCHLOROPHYLLIDE OXIDOREDUCTASE A and B* (*PORA/B)* genes, thereby stimulating their expression (Reinbothe et al. 1996). Additionally, PIF1 serves a comparable function by repressing Pchlide accumulation by directly interacting with the promoter of the *PORC* gene (Huq et al. 2004). Thus, PIF1 cooperates with EIN3/EIL1 to prevent photooxidative damage and enhance cotyledon greening (Zhong et al. 2009). Furthermore, PIF3 prevents photooxidation and facilitate the greening process by repressing the expression of *GLUTAMYL-tRNA REDUCTASE* (*HEMA1)*, *GENOMES UNCOUPLED 4 and 5 (GUN4/5)* (Shin et al. 2009; Stephenson et al. 2009). Additionally, PIF3 directly interacts with EIN3 and forms an interdependent regulatory module to suppress the expression of most *LIGHT-HARVESTING COMPLEX* (*LHC*) genes, thus synergistically impeding the development of chloroplasts (Liu et al. 2017). The B-box protein BBX11 directly upregulates *HEMA1* and *H SUBUNIT of Mg-CHELATASE* (*CHLH)* expression, thus promoting high levels of Pchlide synthesis. However, PIF3 suppresses the expression of *BBX11*, thereby reducing Pchlide synthesis (Job and Datta 2021). Furthermore, during the seedling greening, PIF1/PIF3 and HY5/HYH antagonistically regulate ROS-responsive gene transcription by directly binding to cis-acting elements in the promoters of key genes such as *ASCORBATE PEROXIDASE 2* (*APX2)*, *ZINC-FINGER PROTEIN 10* (*ZAT10)*, *SIGMA FACTOR BINDING PROTEIN 1* (*SIB1)*, and *ERF4* (Chen et al. 2013)*.* EXECUTER1 and 2 (EX1/2) co-activate and interact with *GUN4/5, WRKY18/40* to upregulate ^1^O_2_-responsive genes and trigger the photo-oxidative response (Li et al. 2023).

Once seedlings emerge and are exposed to light, cotyledons gradually unfold and expand. Red light exposure triggers the interaction of phyB and EIN3/EIL1, enhancing their binding to EBF1/EBF2, thereby facilitating the rapid degradation of EIN3 and promoting the opening and expansion of the cotyledons (Shi et al. 2016b). The TCP4 transcription factor activates the *SAUR* family gene *SAUR50*. The accumulated SAUR50 protein interacts with PP2C-D1 and dampens its phosphatase activity, which leads to cotyledon expansion and apical hook opening (Dong et al. 2019; Wang et al. 2020). Similarly, the skotomorphogenesis suppressor DET1 promotes cotyledon opening by destabilizing PIFs (Dong et al. 2014). Moreover, two miPs, namely *miP1a* and *miP1b*, were markedly induced during the light transition, directly interacting with PIF3/EIN3, and suppressing their oligomerization and DNA-binding activities, and thus promoting the apical hook opening and cotyledon unfolding (Wu et al. 2020a). Our recent results show that *BBX32* acts as a positive regulator during seedling de-etiolation, by directly interacting with EIN3, PIF3 and EBF1/2, disrupting the assembly of the SCF^EBF1/2^-EIN3/PIF3 E3 ligase protein complexes, thereby dampening E3 ligase activity and robustly controlling EIN3/PIF3 stability (Wang et al. 2025). Beyond its role in hook development, HLS1 functions as a histone acetyltransferase to promote cotyledon greening. It directly activates greening-related genes by depositing H3K9ac and H3K27ac marks upon light exposure (Peng et al. 2025).

## Deep sowing in crops: mesocotyl and coleoptile regulation

Seedlings from different species employ diverse adaptive strategies to emerge from deep-sowing soil conditions (Sun et al. 2024). Monocots and dicots demonstrate systematic differences in seed structure and employ distinct protective strategies throughout the seedling emergence process. Monocot seeds are characterized by a single, typically small slender cotyledon, that retains a substantial amount of endosperm during embryonic development. The endosperm serves as the principal nutrient reservoir for seed germination. In contrast, dicot seeds possess two larger and fatter cotyledons that store abundant nutrients for seed germination, rendering the endosperm largely redundant or entirely absent in most cases (Sabelli 2012). In monocot plants like maize and rice, the seedling establishment phase lacks the formation of an apical hook and hypocotyl. Upon germination, the mesocotyl and coleoptile emerge from the seed, enclose and protect the primary meristematic tissues, allowing them to break through the soil surface and begin photosynthesis (Niu et al. 2020; Saenz Rodriguez And Cassab 2021). Therefore, mesocotyl and coleoptile elongation is a key adaptive trait under deep-sowing conditions in monocotyledonous plants (Table 1).

**Table 1:** Known genes in crops deep sowing regulation

Gene ID	Name	Functions	References
*Os03g0324200*	*OsEIL1*	Promotes mesocotyl elongation by the ET, GA and ROS pathways	Lyu et al. 2024; Qiao et al. 2024
*Os07g0685700*	*OsEIL2*	Suppresses *OsGY1* and activates antioxidant genes to promote seedling emergence	Xiong et al. 2017; Qiao et al. 2024
*Os07g0155600*	*OsEIN2*	Integrates ET-JA signaling to promote mesocotyl elongation and seedling emergence	Xiong et al. 2017
*Os03g0782500*	*OsPIF4/PIL13*	Enhances the transcriptional activation on *EXPs* genes to show higher emergence rate and longer mesocotyl	Lyu et al. 2024;
*Os01g0883800*	*ME1/SD1*	Promotes mesocotyl elongation via the GA-SLR1-OsPIF4/PIL13 pathway	Lyu et al. 2024
*Os01g0900400*	*OsGY1*	Mediates JA-regulated mesocotyl growth downstream of OsEIN2	Xiong et al. 2017
*Os04g0671300*	*OsPAO5*	Released more H_2_O_2_ and reduced ethylene synthesis to show shorter mesocotyls	Lv et al. 2021
*Os11g0609600*	*OsGF14h*	Promotes flooding tolerance through increased coleoptile length	Sun et al. 2022
*Os11g0446700*	*OsUGT75A*	Enhances shoot elongation during deep sowing	Zhao et al. 2024a, b
*Os05g0207500*	*OsGSK2*	Determine mesocotyl length variation by coordinating strigolactone and brassinosteroid signalling	Sun et al. 2018
*Os01g0711500*	*OsCBL10*	Enhances coleoptile length and survival rate during seed germination under submergence	Ye et al. 2018
*GRMZM2G104920*	*ZmCOP1*	Promotes the elongation of etiolated seedlings and plant height	Chen et al. 2023
*GRMZM2G122543*	*ZmSRO1e*	Physically interacts with ZmbZIP61 and positively regulates mesocotyl elongation	Qin et al. 2024
*GRMZM2G137046*	*ZmbZIP61*	Represses the expression of *ZmEXPB*s to inhibit mesocotyl elongation	Qin et al. 2024
*Zm00001d007107*	*ZmCCT2*	*ZPUM5-ZmCCT2-ZmEXPs/ZmPIFs* module positively regulates mesocotyl elongation and altitude adaptation	Zhang et al. 2025
*Zm00001d031529/Zm00001d049761*	*ZPUM5A/ZPUM5B*	Negative regulates mesocotyl elongation by binding to *ZmCCT2* 3’ UTR and inhibits the protein translation	Zhang et al. 2025

Deep sowing allows seeds with the opportunity to germinate and grow in a more stable soil environment, thereby enhancing seedling emergence and survival rates (Zhao et al. 2021; Rebetzke et al. 2005). The ethylene-responsive transcription factors OsEIL1 and OsEIL2 are indispensable for rice seedling emergence under deep-sowing conditions. OsEIL1 specifically targets the promoter region of *MESOCOTYL ELONGATION1* (*ME1*), promoting GA synthesis. This minimize the supressive impact of DELLA proteins on PIFs, enhancing the transcriptional activation of downstream expansin genes and ultimately promoting mesocotyl elongation (Lyu et al. 2024). OsEIL2 and OsEIN2 repress the transcriptional activity of GAOYAO1 (OsGY1) to reduce JA accumulation and promote mesocotyl and coleoptile elongation (Xiong et al. 2017). OsEIL1/2 directly bind to the promoters of *OsVTC1-3* and *OsPRX37/81/82/88*, activating their expression, stimulating coleoptile elongation to enable seedling emergence (Qiao et al. 2024). Furthermore, OsPAO5 encodes a polyamine oxidase in rice, that plays a pivotal role in polyamine metabolism by oxidizing polyamines to generate hydrogen peroxide, thereby curbing ethylene production and leading to a reduction in mesocotyl length (Lv et al. 2021). The 14–3-3 protein OsGF14h promotes flooding tolerance through increased coleoptile length during germination (Sun et al. 2022). Moreover, UDP-GLUCOSYLTRANSFERASE75A (OsUGT75A) enhances shoot elongation during deep sowing, by promoting cell elongation and shoot length in rice (Zhao et al. 2024b). The mesocotyl and coleoptile can act as selection organs for assessing stress and deep-sowing acclimation during the initial seedling phase (Niu et al. 2020; Sun et al. 2024). Through a genome-wide association study (GWAS), researchers found that diversity in rice mesocotyl length is controlled by natural variation in the OsGSK2 gene, which modulates both strigolactone and brassinosteroid signaling, a key adaptive trait selected during domestication (Sun et al. 2018). Natural variation in a CALCINEURIN B-LIKE PROTEIN 10 (OsCBL10) has been shown to affect coleoptile length and enhance survival during seed germination under submergence (Ye et al. 2018).

The maize mesocotyl rapidly elongates during seed germination in the dark (in soil) and elevating the coleoptile (along with the enclosed plumule) towards the soil surface. Therefore, it is highly related to maize deep-sowing tolerance (Zhao et al. 2010; Zhao and Wang 2008). Research has demonstrated that *ZmCOP1* regulates mesocotyl elongation and plant height in maize through the BR signal transduction pathway (Chen et al. 2023). ZmSRO1e, a member of the plant-specific SIMILAR TO RCD-ONE (SRO) protein family, interacts with ZmbZIP61 and hinders with its transcriptional activity by binding to target gene promoters such as *ZmEXPB4* and *ZmEXPB6*, thereby facilitating mesocotyl elongation (Qin et al. 2024). Overexpression of *ZmCCT2* (*COL SUBFAMILY OF CCT PROTEINS*) upregulates the transcription of *ZmPIFs*, thereby enhancing mesocotyl elongation. Conversely, the RNA-binding proteins ZPUM5A/B interact with the 3′ UTR of *ZmCCT2* and suppress its protein translation (Zhang et al. 2025).

## Perspectives

### Tissue-specific response genes during seed-to-seedling transition will need further elucidation

The seed-to-seedling transition represents the vital stage in the life of seed plants. Under permissive conditions, seeds sense light, absorb water, and expand, with the radicle emerging from the germination pore. The hypocotyl then grows rapidly and pushes the cotyledons above the ground, while true leaves emerge, completing the transformation from seed-to-seedling. This transition undergoes multiple stages, and it is necessary to perform detailed studies to evaluate at which stage the seeds or seedlings die and fail to establish. Furthermore, different organs of seeds and seedlings play distinct roles during this transition. Notably, although PIFs and EIN3 participate in numerous light signaling pathways, their functional outputs are highly dependent on the specific stage of growth and development (Shi et al. 2018; Zhong et al. 2014). Critical questions remain regarding how cell type-specific gene expression patterns, are determined and how they ulter in distinct cells during the seed-to-seedling transition. Advances in single-cell sequencing or spatial transcriptomic technologies will facilitate scientists to zoom in on spatiotemporal patterns related to these questions.

### Light mediated seed-to-seedling transition tap into the mechanical interplay awaits further study

The capacity to perceive and respond to a wide variety of mechanical stimuli, including gravity, touch, osmotic pressure, or the cell wall resistance, represents an essential aspect of plant biology. Mechanoperception and mechanotransduction processes are crucial for ensuring normal growth and development across cellular, tissue, and whole-plant scales, as well as for orchestrating appropriate responses to a range of diverse environmental stresses, both biotic and abiotic (Monshausen and Haswell 2013; Copenhaver et al. 2007). The mechanical impedance caused by the soil overlay quantitatively activates ethylene production in seedlings to induce soil mechanical stress signaling pathway (Goeschl et al. 1966; Zhong et al. 2014; Wu et al. 2020b; Kays et al. 1974). Ethylene pathway mutants display impaired soil emergence, further reinforcing the critical role of ethylene in soil emergence (Harpham et al. 1991; Zhong et al. 2014; Wu et al. 2020b). Soil overlay triggers an ethylene signaling cascade, inducing the classic “triple response” which includes suppression of hypocotyl elongation, promotion of hypocotyl radial expansion, and exaggerated bending of the apical hook, thereby effectively protecting against mechanical injuries and enabling seedlings to penetrate through the soil (Shi et al. 2016a). Recent studies have increasingly evident that ethylene is pivotal for orchestrating plant protrusion from the soil and subsequent greening (Wu et al. 2020b; Shi et al. 2016a; Zhong et al. 2014; Wang et al. 2025).

Although the elucidation of mechanoreceptors remains a significant impediment, researchers are progressively uncovering the evolving commonalities in the early stages of mechanical signal transduction. Physiological investigations have underscored the central role of the secondary messenger Ca^2+^ in numerous mechanotransduction pathways (Yahraus et al. 1995; Monshausen et al. 2009; Felle and Zimmermann 2007). Ascertaining the transporters responsible for the precise shaping of Ca^2^⁺ signatures and the subsequent mediation of downstream ion fluxes are critical for unraveling the generation and interpretation mechanisms of Ca^2+^ signals, potentially providing tools to manipulate Ca^2+^-dependent mechanical responses (Monshausen and Gilroy 2009). The molecular mechanisms that translate mechanical stimuli into cellular responses within plants are still not well understood (Landrein and Ingram 2019). In summary, future research will yield exciting new perception into the molecular mechanisms of mechanotransduction.

Photobiology, plant hormones and mechanical stimulus signaling pathways have generally been studied separately, however, recent research has begun to uncover the crosstalk among these regulators networks. Within a certain depth range, burial depth can provide sufficient soil moisture and mineral nutrients for germination and seedling establishment, offering a protective effect. However, an excessive burial depth can inhibit seed germination and seedling emergence. Although we have just started to understand the molecular mechanism by which seeds or seedlings sense soil depth (Shi et al. 2016a; Briggs 2016; Braam 2005), limited research has been conducted on the molecular interactions between light and mechanical pressure, including COP1, EIN3, EBF1/2 and HLS1 (Shi et al. 2016a, 2016b; Shen et al. 2016). As our understanding of individual light signaling processes and mechanical pathways deepens, further studies are required to elucidate how light signaling pathways tap into the mechanical pressure-induced signaling pathways. Nevertheless, young seedling establishment is a multifaceted trait that is extremely sensitive to extrinsic conditions, including drought, flooding, salinity, and extreme temperatures such as heat, cold, and soil micro-environmental factors. Future research will be necessary to further reveal the molecular mechanism governing seedling emergence under soil coverage and other combined stress conditions.

### Lights coupling mechanical stimulus to optimize crop yields

Most research on the interplay between light and mechanical stimuli has focused on model plants, with crops receiving relatively little attention (Liu and Li 2024). Nevertheless, the simplified cultivation strategy of direct-seeding, especially dry direct seeding for rice, wheat, corn, soybeans and cotton is gradually attracting attention due to its low cost and convenience (Gommers and Monte 2018; Warpeha and Montgomery 2016). However, this approach is prone to issues such as inadequate seedling stands, weed proliferation, and severe lodging risk in the field, all of which directly impact the final crop yield (Gardarin et al. 2016). Conversely, sowing at depth is usually employed to minimize the damage caused by wildlife and to lower the risk of lodging (Gardarin et al. 2016; Liu et al. 2025; Lv et al. 2021; Zhan et al. 2019; Zhao et al. 2024b). Moreover, it has been shown that applying mechanical stimulation to young seedlings can alter their morphological, anatomical, and biochemical traits linked to lodging resistance (Hansen et al. 2025). Deep-sowing tolerant seedlings not only reduce lodging risk, but also successfully emerge from deeper soil layers where moisture conditions are favorable for seed germination. Improving maize emergence from depth is a key breeding target that could confer pleiotropic benefits, including enhanced drought and heat resilience. This integrated stress tolerance is crucial for securing uniform seedling stands and maximizing productivity in variable global environments (Niu et al. 2020).

Therefore, conducting a more thorough screening of key genes and elucidating the precise molecular mechanisms underlying germination and seedling emergence in crops is beneficial for enhancing crop yields. Although the loci regulating deep sowing have been identified, the genetic network and engineering modifications of the deep-sowing-related genes in crops are extremely limited. Future studies conducted with the aid of genetic engineering techniques, including CRISPR-Cas9 editing, are of vital importance for promoting the design of crop varieties with stress resistance, deep-sowing tolerance and high-yielding crops in the near future.

## Data Availability

Not applicable.

## Abbreviations

COP1: CONSTITUTIVE PHOTOMORPHOGENIC1
HY5: ELONGATED HYPOCOTYL5
PIFs: PHYTOCHROME-INTERACTING FACTORS
EIN3: ETHYLENE-INSENSITIVE 3
EIL1: EIN3-LIKE 1
HFR1: LONG HYPOCOTYL IN FAR-RED1
COP10: PHOTOMORPHOGENESIS 10
DET1: DE-ETIOLATED1
EFS: EARLY FLOWERING IN SHORT DAYS
GAI: GIBBERELLIC ACIDINSENSITIVE
RGA: REPRESSOR OF GA1-3
DAG1: DOF AFFECTING GERMINATION1
JMJ20: JUMONJI20
PCY: PLANTACYANIN
HLS1: HOOKLESS1
CTR1: CONSTITUTIVE TRIPLE RESPONSE1
XTHs: XYLOGLUCAN ENDOTRANGLUCOSYLASEs/HYDROLASES
EXPs: α-EXPANSINS
PP2C-D1: PHOSPHATASE 2C D-CLADE 1
BIN2: BRASSINOSTEROID-INSENSITIVE 2
SIZ1: SAP AND MIZ1 DOMAIN-CONTAINING LIGASE1
PGX3: POLYGALACTURONASE INVOLVED IN EXPANSION
PORA/B: PROTOCHLOROPHYLLIDE OXIDOREDUCTASE A and B
EX1: EXECUTRE1
RVE1: REVEILLE1
BBX32: B-BOX DOMAIN PROTEIN 32
ABI3/5: *ABSCISIC ACID INSENSITIVE 3/5*
ABD1: ABA-HYPERSENSITIVE DCAF1 (ABD1)
*SAUR17*: *SMALL AUXIN UP RNA17*
SUMO: Small Ubiquitin-like Modifier
miPs: MicroProteins
Pchlide: Protochlorophyllide
ROS: Reactive Oxygen Species
NO: Nitric Oxide
GA: Gibberellins
ABA: Abscisic acid
AUX: Auxins
ET: Ethylene
JA: Jasmonic acid
BR: Brassinosteroid
CK: Cytokinin
